# AbSeq Protocol Using the Nano-Well Cartridge-Based Rhapsody Platform to Generate Protein and Transcript Expression Data on the Single-Cell Level

**DOI:** 10.1016/j.xpro.2020.100092

**Published:** 2020-08-25

**Authors:** Jami R. Erickson, Florian Mair, Grace Bugos, Jody Martin, Aaron J. Tyznik, Margaret Nakamoto, Stefanie Mortimer, Martin Prlic

**Affiliations:** 1 Fred Hutchinson Cancer Research Center, Vaccine and Infectious Disease Division, Seattle, WA 98109, USA; 2 BD Biosciences, La Jolla, CA 92037, USA; 3 Department of Immunology, University of Washington, Seattle, WA 98195, USA

## Abstract

By including oligonucleotide-labeled antibodies into high-throughput single-cell RNA-sequencing protocols, combined transcript and protein expression data can be acquired on the single-cell level. Here, we describe a protocol for the combined analysis of over 40 proteins and 400 genes on over 10^4^ cells using the nano-well based Rhapsody platform. We also include a workflow for sample multiplexing, which uniquely identifies the initial source of cells (such as tissue type or donor) in the downstream analysis after upstream pooling.

For complete information on the use and execution of this protocol, please refer to Mair et al. (2020).

## Highlights

• Step-by-step protocol for obtaining combined transcript and protein single-cell data
• Protocol includes considerations for data analysis
• Sample multiplexing strategy to reduce batch effects is described
• Discussion of enrichment strategies for rare cell types or human tissues

## Graphical Abstract

**Figure:**
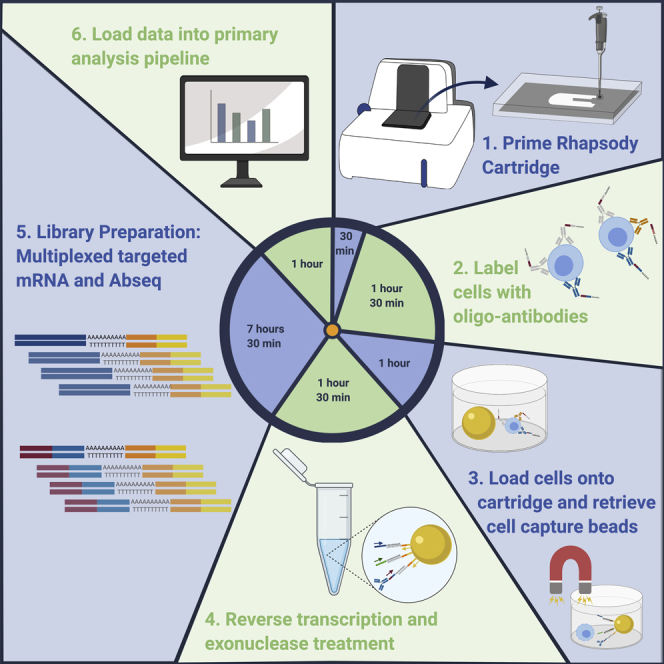

## Before You Begin

This protocol should be read in full prior to starting an experiment. While this protocol may take over 12 h to complete, there are several stopping points that allow experimentation to be segmented over several days if needed. New versions of products discussed within this protocol are continuing to emerge. Ensure you are taking current recommendations on best practices from the product manufacturer.

In addition to standard lab equipment (including a PCR machine, consumables, etc.), access to a Rhapsody Express instrument is needed, as well as Rhapsody-specific reagents including the nano-well cartridges, oligonucleotide-labeled antibodies, library preparation reagents, and a panel of primers targeting the genes of interest. If samples will be multiplexed (optional and described in the protocol), then Sample Tag antibodies are needed as well. A full list of reagents is provided in the Key Resources Table. Furthermore, for quantification and quality control of intermediate and final PCR products a Qubit and TapeStation instrument is required.

As outlined in the protocol, it is critical at several steps to use laboratory practices, reagents, and workspaces that are suitable for working with RNA to avoid contamination with RNAse and subsequent mRNA degradation. Finally, it is essential to carefully consider the number of cells that will be analyzed and, if required, include a cell subset enrichment approach (such as FACS-based purification of cells) prior to starting the workflow. These enrichment techniques can also provide cost savings as AbSeq and mRNA sequencing reads can be limited to the cells of interest. Of note, there are multiple approaches to ensure antibodies used for enrichment do not interfere with oligo-conjugated AbSeq reagents, including choices of non-competing clones. These considerations and others are discussed in more detail in the section “Limitations”. For additional background on the technology underlying surface protein detection using oligo-nucleotide-labeled antibodies, we refer the reader to the following publications (Peterson et al., 2017; Stoeckius et al., 2017).

### Defrosting Cryopreserved Peripheral Blood Mononuclear Cells (PBMCs)

**Timing: 30 min**

1. Prepare media for defrosting cells
a. Warm the following reagents in a 37°C water bath:
i. RPMI 1640 (Thermo Cat # 11875119)
ii. Fetal Bovine Serum (FBS)
iii. L-Glutamine
iv. Penicillin-Streptomycin
b. Make complete media by adding 10% FBS, 1% L-Glutamine, and 1% penicillin-streptomycin to RMPI 1640.
2. Obtain PBMC vials for the desired number of samples from liquid nitrogen.

***Note:*** The sample multiplexing capability is currently limited to a maximum of 12 different samples.

3. Immediately place the PBMC vials into a 37°C water bath.
4. For each vial, remove the PBMCs from the water bath when a small ice pellet remains.
5. Slowly add 1 mL of warm complete media to the PBMCs in the cryovial in a dropwise manner.
6. Transfer the contents of the vial dropwise to a 15 mL conical tube with 10 mL of pre-warmed complete media.
7. Using 1 mL of warmed complete media, rinse the vial to ensure collection of all cells and add to the 15 mL conical tube.
8. Centrifuge cells at 250 × *g* for 5 min.
9. Decant the supernatant.
10. Resuspend pellet for each sample in 5 mL of warm complete media.
11. Count cells using Trypan blue to ascertain cell viability.
12. Take an aliquot containing the desired number of cells for analysis from each sample (it is recommended to start with at least three times the cell number that should be later loaded on the cartridge) and transfer them to new 1.5 mL LoBind tubes.
13. Centrifuge the tubes at 400 × *g* for 5 min and remove the supernatant.
14. Resuspend the cell pellets in 180 μL of Sample Buffer.

***Optional:*** If enrichment of certain cell populations is desired, use a cell sorter following standard practices (Cossarizza et al., 2019) and for each target population collect at least three times the cell number that should be later loaded on the cartridge. Keep sorted cells on ice, wash them after sorting and then resuspend them in 180 μL of cold Sample Buffer.

***Note:*** all subsequent protocol steps can also be used with immune cells isolated from solid tissue samples after sort enrichment, or from fresh peripheral blood samples.

## Key Resources Table

**Table:** 

REAGENT or RESOURCE	SOURCE	IDENTIFIER
**Antibodies**		

BD AbSeq antibody-oligos	BD Biosciences	various

**Biological Samples**		

Cryopreserved peripheral blood mononuclear cells	HIV Vaccine Trials Network, Fred Hutchinson Cancer Research Center	N/A

**Chemicals, Peptides, and Recombinant Proteins**		

Ethyl alcohol, Pure (200 proof, molecular biology grade)	Sigma-Aldrich	E7023-500ML
RPMI 1640	Major Supplier	n/a
Fetal Bovine Serum	Major Supplier	n/a
L-Glutamine	Major Supplier	n/a
Penicillin-Streptomycin	Major Supplier	n/a
Cell Staining Buffer	Major Supplier	n/a
Trypan blue Solution, 0.4%	ThermoFisher Scientific	15250061
70% ethyl alcohol or 70% isopropyl alcohol	Major Supplier	n/a

**Critical Commercial Assays**		

BD Rhapsody Cartridge Reagent Kit	BD Biosciences	633731
BD Rhapsody Cartridge Kit	BD Biosciences	633733
BD Single-Cell Multiplexing Kit – Human Sample Tag 12	BD Biosciences	633781
BD Rhapsody cDNA Kit	BD Biosciences	633732
BD Rhapsody Targeted Amplification Kit	BD Biosciences	633734
BD Rhapsody Targeted Primer Panel	BD Biosciences	various
BD Rhapsody Supplemental Panel	BD Biosciences	633742
Qubit dsDNA HS Assay Kit	Thermo Fisher Scientific	Q32851
SPRIselect Reagent	Beckman Coulter	B23318
High Sensitivity D5000 ScreenTape	Agilent	5067-5592

**Software and Algorithms**		

BD Rhapsody Targeted Analysis Pipeline	SevenBridges	jiewho/bd-public-project/bd-rhapsody-analysis-pipeline
R	The R Project for Statistical Computing	v3.6.3
RStudio	RStudio	v1.2.5042
Seurat	Satija Lab, NYU Genome Center	https://satijalab.org/seurat/

**Other**		

BD Rhapsody Express Single-Cell System	BD Biosciences	633707
6-Tube Magnetic Separation Rack for 1.5 mL tubes	New England Biolabs	S1506S
Large magnetic separation stand	V&P Scientific, Inc.	VP 772FB-1
Clear acrylic cylinder adapter for 15 mL tube magnet	V&P Scientific, Inc.	VP 772FB-1A
Low-profile magnetic separation stand for 0.2 mL, 8-strip tubes	Permagen Labware	MSB08
Themomixer (16°C–37°C, 1,200 rpm)	Eppendorf	5382000023 & 5360000038
Qubit Fluorometer	Thermo Fisher Scientific	Q32866
Heat block capable of 80°C	VWR	10153-348
4200 TapeStation	Agilient Technologies	G2991AA
Thermal cycler with heated lid	Major supplier	n/a
Water bath	Major supplier	n/a
Laminar flow hood	Major supplier	n/a
Digital timer	Major supplier	n/a
Pipettes (P2, P10, P20, P200, P1000)	Major supplier	n/a
Multi-channel pipette, 2–20 μL	Major supplier	n/a
Microcentrifuge for 1.5–2.0 mL tubes	Major supplier	n/a
Microcentrifuge for 0.2 mL tubes	Major supplier	n/a
Centrifuge and rotor for 15 mL tubes	Major supplier	n/a
Vortexer	Major supplier	n/a
Pipet-Aid	Major supplier	n/a
Falcon Tube with Cell Strainer Cap	Thermo Fisher Scientific	352235
Improved Neubauer Hemocytometer	INCYTO	DHC-N01-5
DNA LoBind Tubes, 1.5 mL	Eppendorf	0030.08051
DNA LoBind Tubes, 5 mL	Eppendorf	0030108310
Low retention filtered pipette tips, 10 μL	Major supplier	n/a
Low retention filtered pipette tips, 200 μL	Major supplier	n/a
Low retention filtered pipette tips, 1,000 μL	Major supplier	n/a
Gilson PIPETMAN Tipack Filtered Tips, 100–1,200 μL	Thermo Fisher Scientific	F171803G
Gilson PIPETMAN Tipack Filtered Tips, 500–5,000 μL	Thermo Fisher Scientific	F161370G
Qubit Asssay Tubes	Thermo Fisher Scientific	Q32856
0.2 mL PCR 12-strip tubes	Major supplier	n/a
10 mL sterile serological pipettes	Major supplier	n/a
Premoistened cleaning wipes with 70% ethyl alcohol or 70% isopropyl alcohol	Major supplier	n/a

**CRITICAL:** BD Stain Buffer contains Sodium Azide. Contact with acidic solutions and metal compounds over time may form potentially explosive metal azides. Should any of this material be introduced into a sanitary sewer system, flush with copious amounts of water.

## Materials and Equipment

***Alternatives:*** Agencourt AMPure XP magnetic beads (Beckman Coulter, Cat. No. A63880) can be used instead of SPRISelect Reagent. Instead of the BD Rhapsody Express, the Rhapsody Single-Cell Analysis System (BD Cat. No. 633701) can be used, which provides additional QC steps by imaging cells in the wells of the cartridge. Furthermore, the 2100 Bioanalyzer (Agilient Technologies Cat. No. G2940CA) can be used instead of the 4200 TapeStation. If a Bioanalyzer will be used, High Sensitivity Kit for Bioanalyzer (Aligent 5067-4626) can be used in place of High Sensitivity D5000 ScreenTape. Instead of using the package Seurat (Butler et al., 2018) in R and RStudio, SeqGeq (BD Biosciences) can be used to analyze data. A comprehensive review of analysis packages for single-cell analysis pipelines is provided on https://osca.bioconductor.org/ (Amezquita et al., 2020).

## Step-By-Step Method Details

### Preparing the Cartridge

**Timing: 30 min**

The cartridge is essential for partitioning of single cells. There are two key steps to preparing the cartridge before addition of cells: priming the cartridge and treating the surface. It is possible to run two cartridges in parallel using a single Rhapsody Express instrument.

1. From the Rhapsody cDNA kit (Cat. No. 633773), thaw the following reagents at room temperature (15°C–25°C), then place on ice:
a. Nuclease-free water
b. RT Buffer
c. RT 0.1 M DTT
d. dNTP
e. RNase Inhibitor
f. Bead RT/PCR Enhancer
g. 10× Exonuclease I buffer
h. Bead resuspension buffer

**CRITICAL:** Only remove enzymes from −20°C when in use.

Place on ice:

i. Sample Buffer
j. 1 M DTT
k. Lysis Buffer
l. Cell Capture Beads
m. Bead Wash Buffer

***Note:*** Ensure that the Eppendorf SmartBlock Plate is installed on the thermomixer and is set to 21°C.

2. Set a heatblock or additional thermomixer with the Eppendorf SmartBlock 1.5 mL to 80°C.

**CRITICAL:** All steps performed on the cartridge will only use electronic Gilson pipettes. Regular pipettes cannot be used for these steps, as the rate of flow into the cartridge is carefully set and controlled by the Gilson pipettes to maximize cell and bead retention during loading and wash steps.

**Figure 1:**
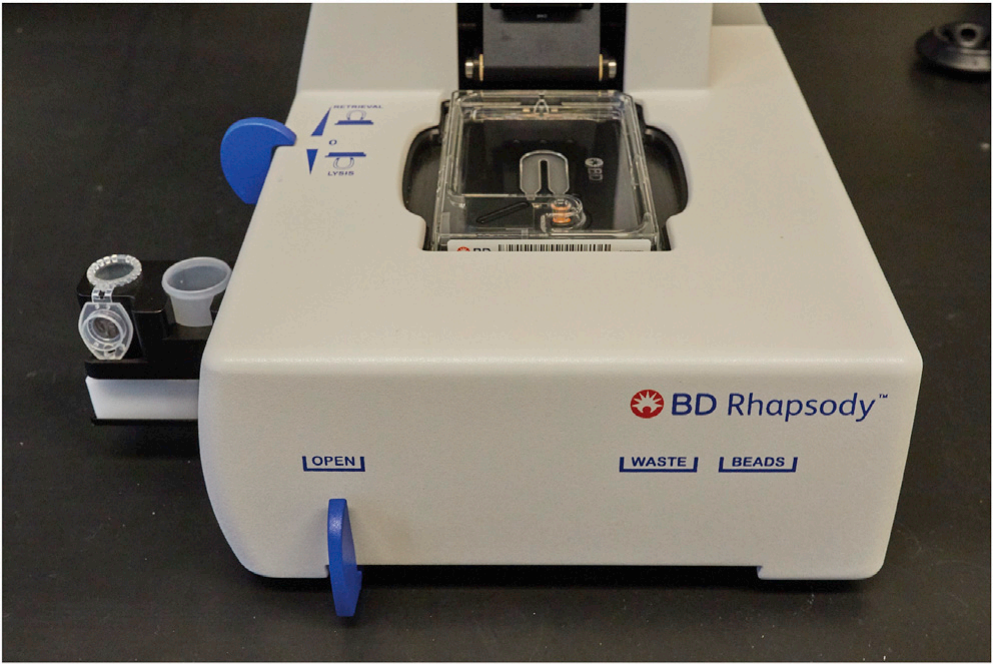
Setting Up the BD Rhapsody Express BD Rhapsody Express showing proper cartridge position with 5 mL LoBind Tube for collection of Cell Capture Beads and waste container installed. During the workflow, the slider needs to be moved either to “Waste” or to “Beads” as indicated in the protocol.

**Figure 2:**
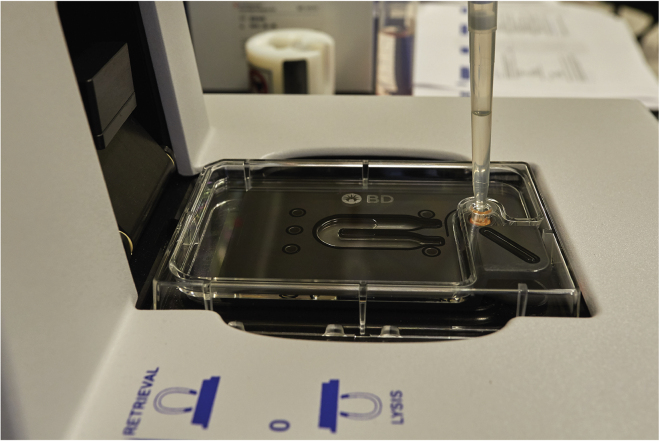
Loading the Cartridge Insert the pipette perpendicular to the cartridge with enough pressure to properly seal the orange gasket with the pipette tip, then dispense the pipette contents. The pipette needs to be set to “Prime/Treat”.

3. Priming the Rhapsody Cartridge
a. Push the cartridge into the far end of the Express instrument tray to match the cartridge and tray notches. Lay the cartridge flat and release it. Ensure that the cartridge is flat in the tray and the barcode faces out.
b. Move the left slider to the middle (0) position on the Express instrument. The Retrieval (top) magnet and Lysis (bottom) magnets are away from the cartridge tray.
c. Move the front slider to **OPEN**
d. Remove the cap of a waste collection container (PN 650000090) and insert both the container and a new 5 mL LoBind Tube (Eppendorf cat. no. 0030108310) for bead retrieval into the appropriate slots in the drawer. Secure the cap of the 5 mL LoBind Tube to the holder (Figure 1).

e. Move the front slider to **WASTE**
f. Insert the tip of the pipette perpendicular to the port, seal the pipette tip against the gasket, and then load the cartridge with 700 μL of 100% (absolute) ethyl alcohol using the Gilson P1200M pipette in **Prime/Treat** mode (Figure 2).

**CRITICAL:** ethyl alcohol should be steadily flowing through the cartridge. If ethyl alcohol is not going into the cartridge, the amount of pressure on the gasket from the pipette needs to be modified to allow reagent flow.

g. Load the cartridge with 700 μL of air using the Gilson P1200M pipette in **Prime/Treat** mode.
h. Load the cartridge with 700 μL of Cartridge Wash Buffer 1 (PN 650000060) with the Gilson P1200M pipette in **Prime/Treat** mode (Figure 2).
i. Leave the cartridge on the tray at 15°C–25°C for 1 min.
4. Treating the surface of the cartridge
a. Load the cartridge with 700 μL of air using the Gilson P1200M pipette in **Prime/Treat** mode.
b. Load the cartridge with 700 μL of Cartridge Wash Buffer 1(PN 650000060) using the Gilson P1200M pipette in **Prime/Treat** mode (Figure 2).
c. Leave the cartridge on the tray at 15°C–25°C for 10 min.

***Note:*** If using two cartridges with a single instrument for one experiment, start the priming/ treating of the second cartridge during this 10-min incubation and repeat the steps above to prepare the second cartridge.

d. Load the cartridge with 700 μL of air using the Gilson P1200M pipette in **Prime/Treat** mode.
e. Load the cartridge with 700 μL of Cartridge Wash Buffer 2 (PN 650000061) using the Gilson P1200M pipette in **Prime/Treat** mode (Figure 2).

***Note:*** The cartridge can be stored at 15°C–25°C for ≤4 h. You can leave the cartridge on the tray. The performance of the cartridge has not been validated at 15°C–25°C storage for >4 h.

### Perform Sample Multiplexing (Optional) and Ab-Oligo Staining

**Timing: 1.5 h**

This protocol describes use of antibodies conjugated to oligonucleotides (Ab-Oligo) for sample multiplexing and surface protein profiling. Each Ab-Oligo contains an antibody-specific barcode, a poly(A) tail for bead capture and additional sequences for PCR amplification and library generation. There are two different methods to utilize multiplexing and surface protein profiling: co-labeling or sequential labeling. Co-labeling will save time, however, sequential labeling is more economical and can additionally reduce batch effects resulting from inconsistent staining. The following steps describe sequential Ab-Oligo labeling of 20,000–1 million cells. Up to 100 antibodies can be pooled together per staining reaction. If no sample multiplexing is required, proceed directly to step 8 (Fc receptor blocking).

5. Use the cell suspensions derived from different samples (or from sorted populations) from the “Defrosting cryopreserved Peripheral Blood Mononuclear Cells (PBMCs)” section, and make sure that each sample is resuspended in 180 μL of Cell staining Buffer.
6. Labeling samples with sample multiplexing antibodies
a. Prepare a spreadsheet listing which sample is going to be labeled with which Sample Tag (1–12).
b. Quick-spin Sample Tag tubes to collect the contents at the bottom.
c. For each sample, transfer 180 μL cell suspension to the corresponding Sample Tag tube. Pipette-mix.
d. Incubate at room temperature (15°C–25°C) for 20 min.
e. Transfer each labeled cell suspension to a 1.5 mL LoBind tube.
f. Add 1 mL Cell Staining Buffer to labeled cells and pipette-mix.
g. Centrifuge each tube at 400 × *g* for 5 min.
h. Pipette off the supernatant taking care to not disturb the cell pellet.

***Note:*** Pipetting off supernatant as opposed to decanting will reduce cell loss during washes. However, it is critical to wash the cells well (i.e., leave as little supernatant as possible).

i. Repeat steps 6f–6h for a total of three washes
j. Resuspend the cell pellet cells in 100 μL cold Cell staining buffer
7. Pooling the samples after labeling with multiplexing antibodies

***Optional:*** if the individual cell samples were obtained from different sources and the cell concentration is unknown, count the cells from step 6j using Trypan blue to determine the mixing ratio as required.

a. Pool all the individual samples from 6j into a new 1.5 mL LoBind tube.
b. Add additional Sample Buffer to bring the total volume to 1.5 mL.
c. Centrifuge the tube at 400 × *g* for 5 min.
d. Pipette off the supernatant taking care to not disturb the cell pellet.
e. Resuspend the cells in 100 μL cold staining buffer.

***Note:*** Samples containing myeloid and B cells should be treated with Fc-blocking reagent prior to Ab-Oligo staining (Andersen et al., 2016).

8. Blocking non-specific Fc Receptor Ab-Oligo Binding.
a. Pipette reagents into a new 1.5 mL LoBind tube on ice (Table 1):

Reagents should be added sequencially, as listed in the table, into a 1.5 mL LoBind Tube on ice. Mix can be scaled according to number of samples.

b. Pipette-mix Fc Block master mix, and briefly centrifuge.
c. Add 100 μL Fc block master mix to the cells from step 7e.
d. Incubate cells on ice for 10 min
e. Add 1 mL of cold staining buffer to wash
f. Centrifuge the tube at 400 × *g* for 5 min
g. Pipette off the supernatant taking care to not disturb the cell pellet
h. Resuspend the cells in 100 μL cold staining buffer.

***Note:*** When handling low cell numbers, the washing steps 8e–h can be omitted.

9. Preparing 2× BD AbSeq antibody-oligo labeling master mix on ice
a. Centrifuge BD AbSeq Ab-Oligos in a tabletop centrifuge at 400 × g for 30 s and place on ice.

***Note:*** Alternatively**,** Ab-Oligos can be placed into a Latch Rack for 500 μL Tubes (ThermoFisher Scientific, Cat. No. 4890) on ice and be centrifuged in the Latch Rack with a plate adapter. Further, tubes can be uncapped and re-capped with an 8-Channel Screw Cap Tube Capper (Thermo Fisher Scientific Cat. No. 4105MAT) and aliquoted with a multi-channel pipette.

b. In pre-amplification workspace, pipette reagents into a new 1.5 mL LoBind Tube on ice (Table 2):

Master mix can be scaled based on number of samples.

c. Pipette-mix the 2× AbSeq labeling master mix, and place back on ice.

***Note:*** The final working concentrations for the Ab-Oligos are between 0.1 μg and 1 μg per stain depending on the antibody clone and have been optimized by the manufacturer. For an example of final working concentrations on a select set of Ab-Oligos, see Supplemental Table 4 in (Mair et al., 2020).

10. Labeling samples with AbSeq Ab-oligos
a. In a new 1.5 mL LoBind tube, combine 100 μL pooled cell suspension labeled with Sample Tags (obtained from step 7e or 8h) and 100 μL 2× AbSeq labeling master mix. Pipette-mix.
b. Incubate on Ice for 30 min.
11. Washing Labeled Cells
a. Add 1 mL Cell staining Buffer to labeled cells and pipette-mix.
b. Centrifuge the tube at 400 × g for 5 min.
c. Pipette off the supernatant taking care to not disturb the cell pellet.
d. Repeat steps 11a–c for a total of 3 washes.
e. Resuspend pellet in 100 μL cold Sample Buffer and use 10 μL to perform a Trypan blue viability staining. Ideally, if planning to load a full cartridge, the cell count should be >300,000 cells/mL with a viability >90%.

**CRITICAL:** Cells must be resuspended in Sample Buffer (and not Staining Buffer)

***Note:*** Sufficient post-labeling washes are important for reducing noise that comes from residual unbound antibodies being captured onto 3’ capture beads during single-cell capture. However, some cell loss occurs with each additional wash. Users can choose to perform more or fewer washes depending on the abundance of their sample. If the user has an excess of cells for the experiment, it is recommended that the stain and wash steps occur in a 5 mL polystyrene tube. This will allow an increase in wash volume to 3 mL instead of 1 mL.

**Table 1:** 2× Fc Block Master Mix

Component	1 Sample (μL)	1 Sample + 20% Overage (μL)
Cell staining buffer	90.0	108.0
Human Fc Block (Cat. No. 5642220)	10.0	12.0
Total	100	120.0

**Table 2:** 2× AbSeq labeling Master Mix

Component	1 Sample (μL)	1 Sample + 10% Overage (μL)	2 Samples + 10% Overage (μL)
Per ab-oligo	2.0	2.2	4.4
Stain Buffer (N= no. ab-oligos)	100.0 - (2.0 ∗ N)	110 - (2.2 ∗ N)	220 – (4.4 ∗ N)
Total	100	110	220

### Loading of Cells onto the Cartridge and Retrieval of Cell Capture Beads Containing mRNA and Feature Barcodes

**Timing: 1 h**

This protocol describes the steps to isolate and capture mRNA and feature barcodes from partitioned cells on the Rhapsody cartridge. Single-cell partitioning is achieved by loading cells at densities that are low enough to achieve a single cell per well distribution in the cartridge. Each cartridge contains >200,000 nanowells, thus the likelihood of having more than one cell in a single well depends on the cell density (see table below). There are different approaches to identify multiplets based on factors such as number of genes or unique molecular identifiers present compared to the rest of the sample, as well as computational approaches (Amezquita et al., 2020). Identifying multiplets can be significantly improved if several samples are multiplexed prior to loading on the cartridge because any sample with two or more Sample Tags is easily identified as a multiplet (Stoeckius et al., 2018).

Refer to Table 3 to determine an acceptable multiplet rate for the number of captured cells on retrieved Cell Capture Beads:

**Table 3:** Expected Multiplet Rate for a Given Cell Load

Number of Captured Cells on Retrieved Cell Capture Beads (Target)	Estimated Multiplet Rate (%)
100	0.0
500	0.1
1,000	0.2
5,000	1.2
10,000	2.4
20,000	4.7

An approximation of the percentage of multiplets that will occur within a cartridge based on the number of cells loaded. Because there are a fixed number of nanowells in a cartridge, the likelihood of multiplets increases with the number of loaded cells. As discussed in the main text, multiplets can only efficiently be removed when using sample multiplexing.

12. According to the number of cells counted in step 11e, take the appropriate volume containing the targeted cell number + 30% (i.e., if planning to capture 20,000 cells per cartridge, take 26,000 cells. If planning to load two cartridges in parallel take 52,000 cells).

***Note:*** 30% is an approximate number that should allow a more accurate prediction of the number of cells collected after. This number takes into account cell loss from both washes and cartridge loading (only 575 μL of the 650 μL single-cell suspension will be loaded onto the Rhapsody Cartridge).

13. Add Sample Buffer to bring the volume of the cell suspension to 650 μL (or 1,300 μL if planning to load two cartridges in parallel)
14. Load the cells onto the cartridge.
a. Load the cartridge on the tray with 700 μL of air using the Gilson P1200M pipette in **Prime/Treat** mode.
b. Change the mode of the Gilson P1200M pipette to **Cell Load.**
c. With a manual pipette, gently pipet the cell suspension up and down to mix.
d. On the Gilson P1200M, press the pipette button once to aspirate 40 μL of air, immerse the pipette tip in cell suspension, and then press the button again to aspirate **575** μL of cold cell suspension (Figure 2).
e. Insert the tip of the pipette perpendicular to the port, seal the pipette tip against the gasket, and then press the button a third time to dispense 615 μL of air and cells.
f. Incubate at room temperature (15°C–25°C) for 15 min. During the 15-min incubation, prepare Cell Capture Beads.

**CRITICAL:** If planning to load two cartridges at the same time using only one Rhapsody Express instrument, load cartridge #2 at the 14-min mark of step 14f by repeating steps 14a–f. This is essential to ensure you have correct timing for subsequent steps.

15. Preparing Cell Capture Beads

***Note:*** Keep the Cell Capture Beads on ice before use. For maximum recovery, do not vortex samples containing Cell Capture Beads. Gently mix suspensions with Cell Capture Beads by pipette only. Use low retention pipette tips and LoBind tubes only.

**Figure 3:**
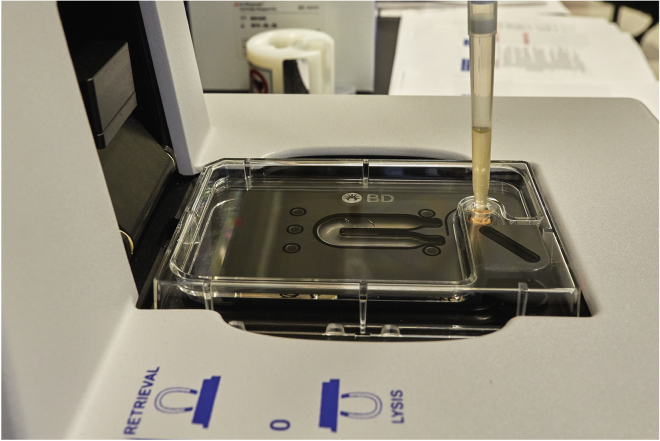
Loading the Cell Capture Beads Hold pipette vertically and with enough pressure to seal the gasket while quickly dispensing beads to avoid settling. The pipette needs to be set to “Bead Load”.

a. Place Cell Capture Bead tube on magnet for 1 min, then remove storage buffer.
b. Remove tube from magnet, and pipette 750 μL cold Sample Buffer into the tube.
c. Pipette-mix and place on ice.
16. Loading the Cell Capture Beads
a. Change the mode of the Gilson P1200M pipette to **Prime/Treat.**
b. At the end of the 15 cell-load incubation, load the cartridge with 700 μL of air using the P1200M pipette in **Prime/Treat** mode.
c. Change the mode of the Gilson P1200M pipette to **Bead Load.**
d. Use a P1000 standard pipette to gently pipet the Cell Capture Beads in cold Sample Buffer (PN 650000062) up and down to mix, and, using the Rhapsody P1200M pipette in **Bead Load** mode, immediately load the cartridge with 630 μL of beads (Figure 3).
e. Let the beads settle in the cartridge on the tray at room temperature (15- 25°C) for 3 min.
f. Place cartridge on the Eppendorf SmartBlock Plates.
g. Shake the cartridge at room temperature (21°C) for 15 s at 1,000 rpm.

**CRITICAL:** Do not change timing and use only SmartBlock Plate for shaking.

h. Blot outlet drip with lint-free wipe.
i. Return cartridge to Express instrument and wait 30 s.
j. Change the mode of the Gilson P1200M pipette to **Wash.**
k. Load the cartridge with 700 μL of air using the Gilson P1200M pipette in **Wash** mode.
l. Load the cartridge with 700 μL of cold Sample Buffer using the Gilson P1200M pipette in **Wash** mode (Figure 2).
m. Repeat steps 16k and 16l once for a total of two washes.
n. Replace Eppendorf SmartBlock Plates adapter with Eppendorf SmartBlock 1.5 mL adaptor and heat thermoblock to 37°C.
17. Lysing the cells
a. Add 75.0 μL of 1 M DTT (PN 650000063) to one bottle of 15 mL Lysis Buffer (PN 650000064), and then check the **Add DTT** box on the Lysis Buffer label. **The Lysis Buffer with DTT must be used within 24 h.**
b. Briefly vortex the lysis mix and place it on ice.
c. Move the left slider to **LYSIS.** The (bottom) magnet is now in the up position and is in contact with the cartridge.
d. Change the mode on the Rhapsody P1200M pipette to **Lysis.**
e. Load the cartridge with 550 μL of Lysis Buffer with DTT using the Rhapsody P1200M pipette in **Lysis** mode (Figure 2).
f. Leave the cartridge on the tray at 15°C–25°C for **2 min**.

**CRITICAL:** If cell lysis step exceeds 2 min, noise from diffusion of transcripts into neighboring wells can increase.

18. During 2-min lysis step:
a. Ensure that a 5 mL LoBind Tube (Eppendorf cat. no. 0030108310) was inserted into the drawer for bead retrieval.
b. Set the mode on the Gilson P5000M pipette to **Retrieval.**
c. Move the front slider to **BEADS.**

**CRITICAL:** If the slider is not set to BEADS, then sample will go into the waste and the experiment is lost.

**Figure 4:**
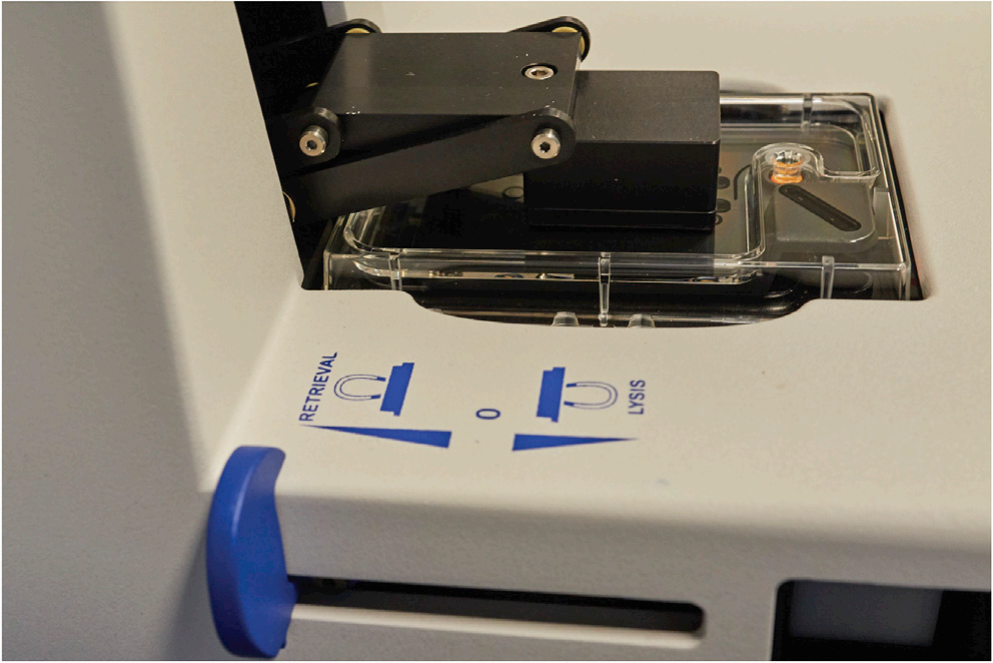
BD Rhapsody Set to Retrieval When the left slider is set to RETRIEVAL, the top magnet will sit atop the cartridge.
b. Leave the Retrieval magnet in the down position for 30 s.
c. During these 30 s, use the Gilson P5000M pipette to aspirate 5,000 μL of Lysis Buffer with DTT.
d. At the end of the 30 s, press down on the Gilson P5000M pipette to seal the pipette tip against the gasket of the cartridge to avoid leaks (Figure 5).

19. Retrieving the Cell Capture Beads from the cartridge
a. Move the left slider to **RETRIEVAL.** The (top) magnet is now in the down position and is in contact with the cartridge (Figure 4).

**Figure 5:**
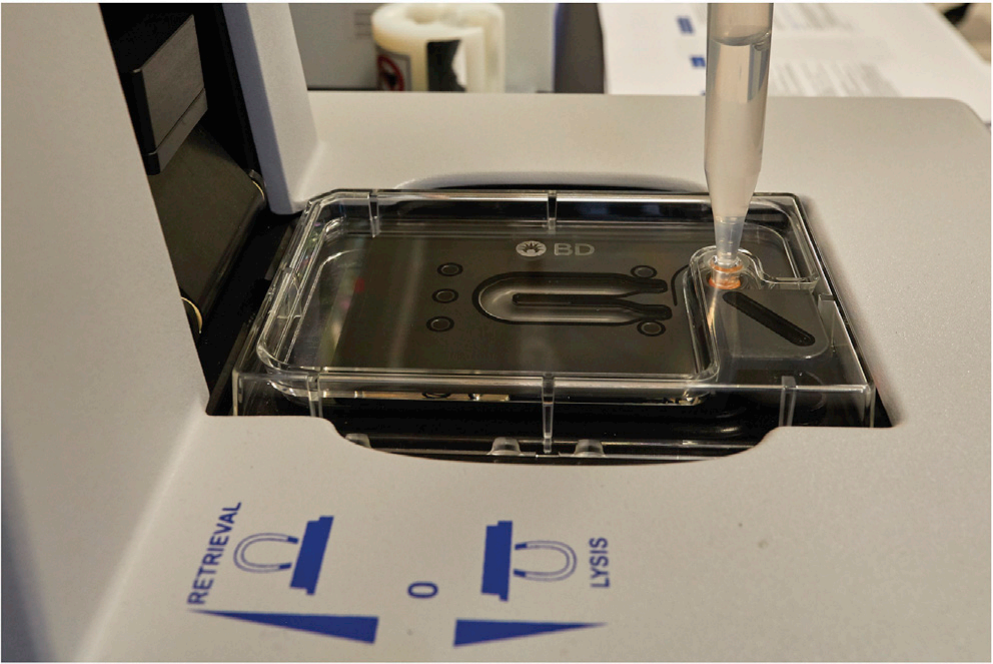
Bead Collection After cell lysis, collect the mRNA and oligo-bound beads using the P5000M pipette. Make sure that the magnet is in the correct position and the tube slider is set to “Beads”.
e. Move the left slider to the middle (**0**) position, and *immediately* load the cartridge with 4,950 μL of Lysis Buffer with DTT using the Gilson P5000M pipette. The Retrieval (top) magnet is in its full up position and is away from the cartridge. The Cell Capture Beads bound to the captured mRNA and feature barcodes are flushed through the cartridge and collected in the 5 mL LoBind tube.
f. Remove the pipette tip from the inlet gasket of the cartridge before pressing the dial button once to purge the tip. Discard the pipette tip.
g. Move the front slider to **OPEN**, and then remove and cap the 5 mL LoBind Tube.
h. Uncap the tube and place it on the large magnetic separation stand fitted with the 15 mL tube adapter for 1 min. Proceed immediately to Washing Cell Capture Beads.
i. Appropriately dispose of cartridge, waste collection container, and Lysis Buffer with DTT.
20. Washing Cell Capture Beads
a. After 1-min incubation leaving the 5 mL tube containing retrieved Cell Capture Beads on large magnet, remove all but 1 mL of supernatant without disturbing beads (Figure 6).

**Figure 6:**
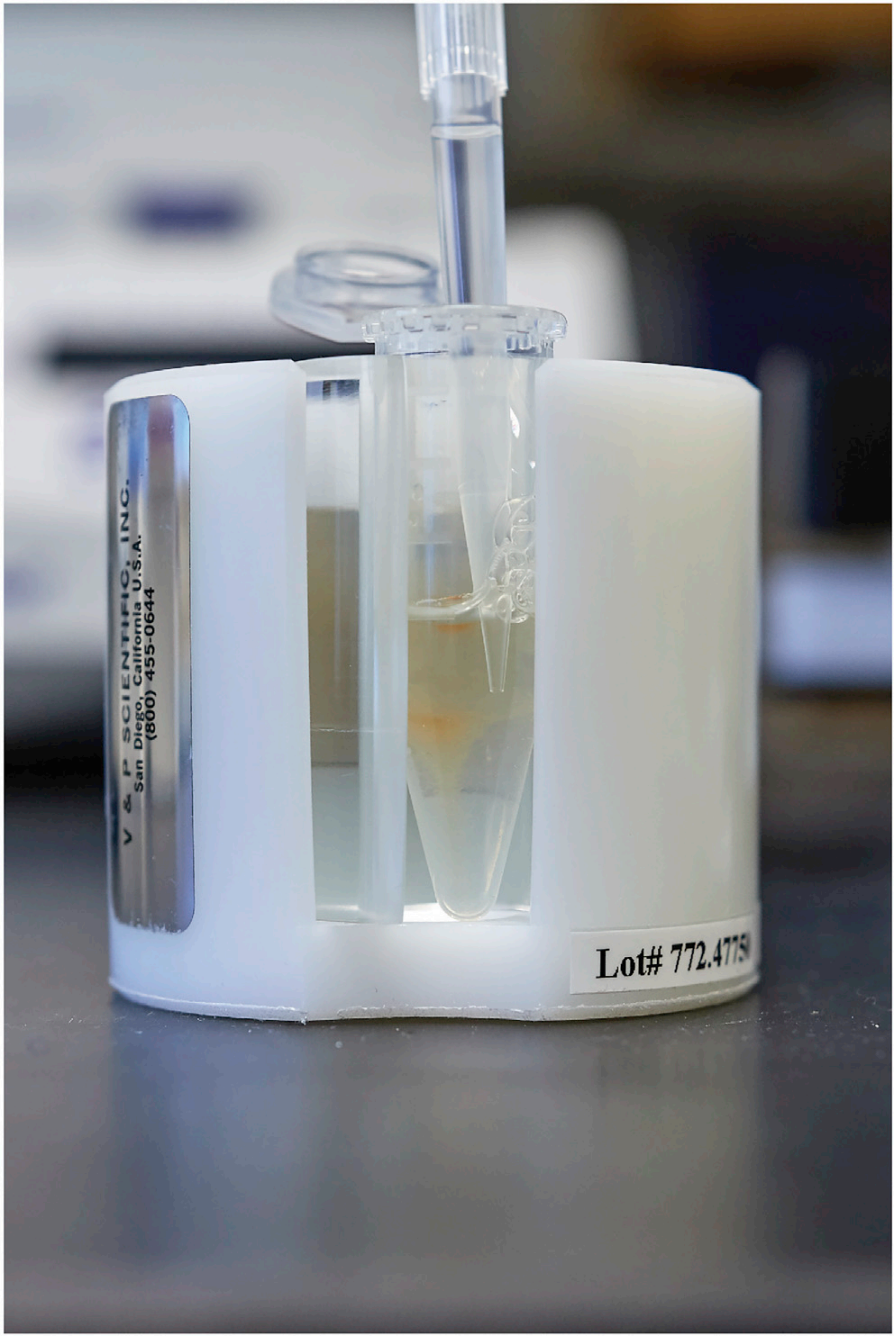
Washing Cell Capture Beads Allow beads to settle next to the magnet, and remove lysis buffer without disturbing the beads.
b. Remove tube from magnet. Gently pipette-mix beads, and transfer to a new 1.5 mL LoBind tube.
c. If there are still beads left in the 5 mL tube, add 0.5 mL Lysis Buffer with DTT, rinse 5 mL tube, and transfer to 1.5 mL LoBind tube from step 20b.
d. Place tube on magnet for ≤ 2 min and remove supernatant.

**CRITICAL:** Avoid leaving Lysis Buffer or bubbles in tube. Lysis Buffer might cause the reverse transcription reaction to fail.

e. Remove tube from magnet and pipette 1 mL of cold Bead Wash Buffer into tube. Pipet mix.
f. Place tube on 1.5 mL tube magnet for ≤2 min and remove supernatant.
g. Remove tube from magnet, and pipette 1 mL cold Bead wash Buffer into tube. Pipette-mix, and place on ice.

**CRITICAL:** Start reverse transcription ≤30 min after washing retrieve Cell Capture Beads with Bead Wash Buffer. If a second cartridge is used, then repeat steps 17a–20g with the second cartridge within this time.

### Performing Reverse Transcription and Exonuclease I Treatment on the Cell Capture Beads

**Timing: 90 min**

This protocol describes the steps to link cell barcodes to mRNA and feature barcodes by reverse transcription, which yields a stable sample and preserving single-cell information for downstream library generation steps. If two cartridges were used in parallel, the two sets of Cell Capture Beads should be kept separate and be treated as two independent libraries.

21. Performing reverse transcription
a. In the pre-amplification workspace, in a new 1.5 mL LoBind Tube that is on ice, pipette the components in the following order to prepare the cDNA mix (Table 4):

Add reagents sequencially, as listed in table, into a 1.5 mL LoBind tube on ice. Volumes can be further scaled up if more than 2 cDNA libraries need to be produced.

b. Gently vortex and centrifuge the mix, and then put it back on ice.
c. Place the tube of washed beads on the 1.5 mL tube magnet for ≤2 min, and then carefully remove and appropriately discard the supernatant without disturbing the beads while leaving the tube on the magnet.
d. Use a low retention tip to pipette 200 μL of the cDNA mix to resuspend the beads. Gently mix the suspension by pipette only. Do not vortex.
e. Transfer the bead suspension to a new 1.5 mL LoBind Tube.
f. Ensure that the SmartBlock Thermoblock 1.5 mL or equivalent is installed on the thermomixer.
g. Incubate the suspension on the thermomixer at 1,200 rpm and 37°C for 20 min.
h. After incubation, put the tube on ice.
22. Treating the Cell Capture Beads with Exonuclease I
a. In the pre-amplification workspace, prepare the Exonuclease I mix in a new 1.5 mL LoBind Tube that is on ice by adding the components in the following order (Table 5):

**Table 4:** cDNA Master Mix

Component	1 Library (μL)	1 Library + 20% Overage (μL)	2 Libraries + 10% Overage (μL)
Nuclease-Free Water	106.7	128.0	235.0
RT Buffer	40.0	48.0	88.0
dNTP	20.0	24.0	44.0
RT 0.1 M DTT	10.0	12.0	22.0
RT/PCR Enhancer	3.3	4.0	7.3
RNase Inhibitor	10.0	12.0	22.0
Reverse Transcriptase	10.0	12.0	22.0
Total	200.0	240.0	440.0

Into a 1.5 mL LoBind tube on ice, add reagents sequencially, as listed in table. Exonuclease I mix can be scaled up to accommodate more than two samples.

b. Gently vortex and centrifuge the mix, and then put it back on ice.
c. Place the tube of beads with cDNA mix on the 1.5 mL tube magnet for ≤2 min, and then carefully remove and appropriately discard the supernatant without disturbing the beads and while leaving the tube on the magnet.
d. Remove the tube from the magnet, and then use a low retention tip to pipette 200 μL of Exonuclease I mix to the tube, gently resuspend the beads by pipette only. Do not vortex.
e. Incubate the suspension on the thermomixer at 1,200 rpm and on the thermomixer at 1,200 rpm and 37°C for 30 min.
23. Inactivating Exonuclease I
a. Transfer the bead suspension with Exonuclease I to the thermomixer or heat block in the pre-amplification workspace at 80°C (no shaking) for 20 min.

***Note:*** If the thermomixer is the same as that used for the 37˚C step put the samples on ice until that temperature is reached rather than leaving the tubes in the thermomixer as the temperature ramps up.

b. Put the bead suspension on ice for ∼1 min.
c. Place the tube on the 1.5 mL tube magnet until the solution is clear (≤1 min).
d. Carefully remove and appropriately discard the supernatant without disturbing the beads while leaving the tube on the magnet.
e. Remove the tube from the magnet, and with a low retention tip, pipette 200 μL of cold Bead Resuspension Buffer to gently resuspend the beads. Do not vortex.

**Pause Point:** The Exonuclease I-treated beads can be stored at 2°C–8°C for ≤3 months.

**Table 5:** Exonuclease I Mix

Component	1 Library (μL)	1 Library + 20% Overage (μL)	2 Libraries + 10% Overage (μL)
Nuclease-Free Water	170.0	204.0	374.0
10 Exonuclease I Buffer	20.0	24.0	44.0
Exonuclease I	10.0	12.0	22.0
Total	200.0	240.0	440.0

### Library Preparation – Multiplexed Targeted mRNA, AbSeq, and Sample Tags

**Timing: 7 h 30 min**

**Figure 7:**
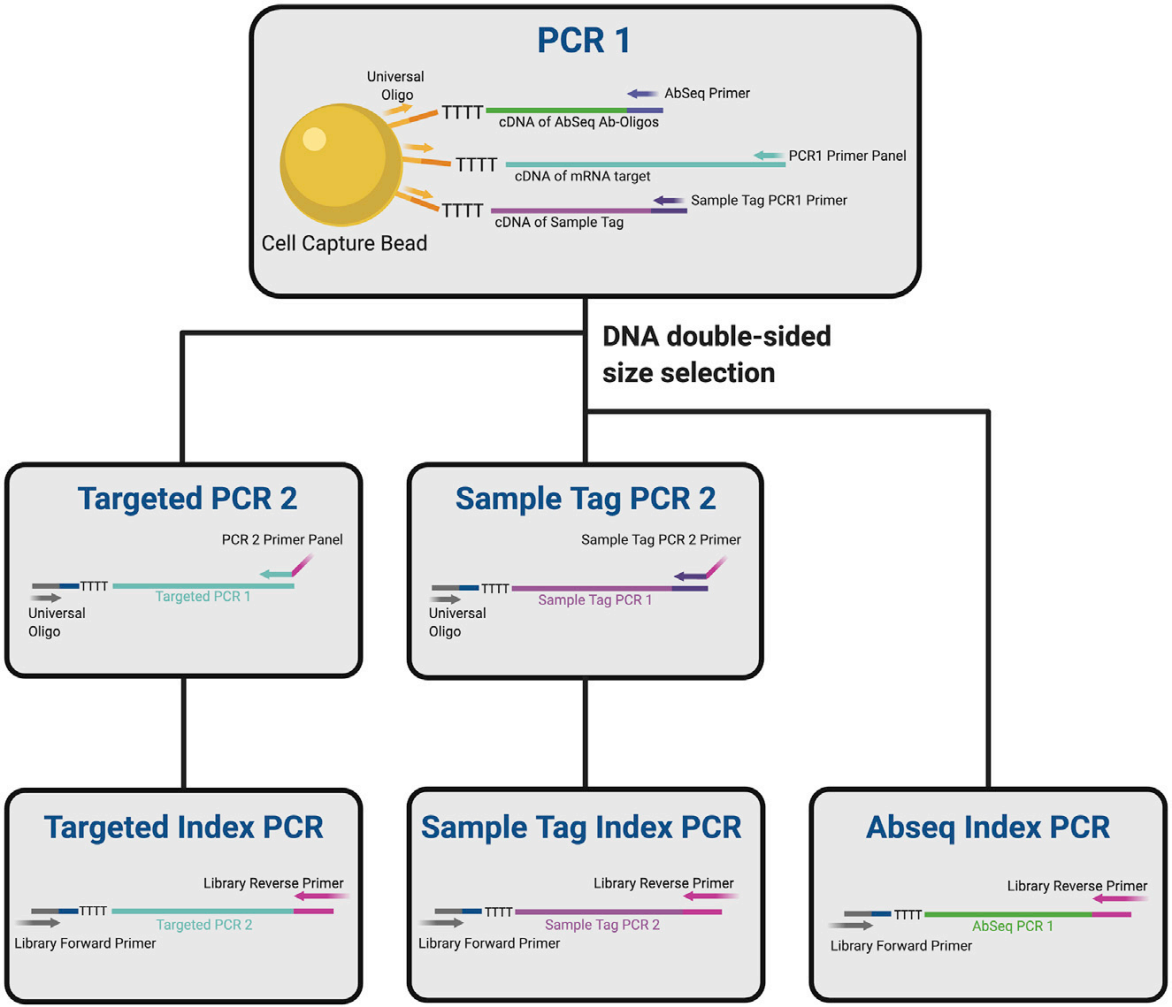
Library Preparation Workflow Overview of each PCR required for library preparation. After PCR1, DNA double-sided size selection will split the transcripts based on amplicon size into mRNA or Sample Tag/AbSeq libraries. PCR2 is then preformed to further enrich both mRNA and Sample Tag libraries. Finally, each library is indexed for sequencing.

This section describes the steps for PCR amplification of the gene targets of interest and the captured features barcodes, as well as the fragment size selection to separate the mRNA library from the AbSeq library and Sample Tag libraries (Figure 7). Different gene panels, including custom gene sets can be used depending on the experimental question.

24. Thaw the following reagents at room temperature (15°C–25°C) and then place on ice:
a. Nuclease-Free water
b. Bead RT/PCR enhancer
c. Elution Buffer
d. Universal Oligo
e. Library Forward Primer
f. Library Reverse Primer 1–4 (depending on what reverse primer desired)
g. PCR1 Panel Supplement (optional)
h. PCR2 Panel Supplement (optional)
i. Bead Resuspension Buffer
j. Sample Tag PCR1 Primer
k. Sample Tag PCR2 Primer
l. BD AbSeq Primer

***Note:*** Only remove enzymes from −20°C when in use.

25. Perform PCR1
a. In pre-amplification workspace, pipette reagents into a new 1.5 mL LoBind Tube on ice (Table 6):

Combine all components into a 1.5 mL LoBind tube on ice. If more than 2 PCR reactions are needed, volumes can be scaled accordingly.

b. Gently vortex mix, briefly centrifuge, and place back on ice.
c. Place tube of Exonuclease I-treated beads in Bead Resuspension Buffer (Cat. No. 650000066) on 1.5 mL magnet for <2 min. Remove supernatant.
d. Remove tube from magnet and resuspend beads in 200 μL PCR1 reaction mix. Do not vortex.
e. Ensuring that the beads are fully resuspended, pipette 50 μL PCR1 reaction mix with beads into each of four 0.2 mL PCR tubes.
f. Bring reaction mix to the post-amplification workspace.
g. Program the thermal cycler (Table 7):

Depending on the number of cells targeted, a different number of cycles should be used to ensure sufficient quantity of library and limit PCR bias. This table is based on resting PBMCs, and should be adjusted accordingly for different cell types. Note that the targeted cell number is lower than the actual number of cells loaded on the cartridge (as calculated in step 12).

h. Ramp heated lid and heat block of post-amplification thermal cycler to ≥95°C by starting the thermal cycler program and then pausing it.
i. For each 0.2 mL PCR tube, gently pipette-mix, immediately place tube in thermal cycler, and unpause the thermal cycler program.

Program a thermocycler to run the above steps. See Table 8 for suggested number of cycles. Do not use fast cycling mode.

**CRITICAL:** The thermocycler should be at 95°C when the tubes are added to ensure amplification of on-bead molecules.

**Pause Point:** Once the PCR1 cycle is complete, the reaction can be stored at 4°C. Purification of PCR1 must occur ≤24 h after PCR1.

j. After PCR, briefly centrifuge tubes.
k. Pipette-mix and combine the four reactions into a new 1.5 mL LoBind Tube.
l. Place the 1.5 mL tube on magnet for 2 min and carefully pipette the **supernatant** (PCR1 products) into a new 1.5 mL LoBind Tube without disturbing the beads.

***Note:*** (Optional) Remove tube with the Cell Capture Beads from magnet, and pipette 200 μL cold Bead Resuspension Buffer into tube. Pipette-mix. Do not vortex. Store beads at 2°C–8°C in post-amplification workspace. Beads can be sub-sampled or undergo multiple rounds of amplification with different primer panels.

26. Perform double-sided SPRISelect bead purification to separate the shorter AbSeq and Sample Tag PCR1 products (∼170 bp) from the longer mRNA targeted PCR1 products (350–800 bp).
a. Prepare 5 mL fresh 80% (v/v) ethyl alcohol by combining 4.0 mL absolute ethyl alcohol, molecular biology grade (major supplier) with 1.0 mL nuclease-free water (major supplier). Vortex tube for 10 s.
b. Vortex SPRISelect Reagent at high speed 1 min until beads are fully resuspended.
c. Pipette 140 μL SPRISelect beads into the tube with 200 μL PCR1 products (step 25l). Pipette-mix 10 times.

**CRITICAL:** SPRISelect beads should be pipetted very carefully. To ensure appropriate size selection, it is essential that only the exact amount of SPRISelect beads is added to PCR1 products. Do not immerse pipette tips with SPRISelect bead droplets on the outside into PCR1 products. Instead, place tip on side of tube and slowly expel all liquid, replace pipette tip, and pipette-mix.

d. Incubate at room temperature (15°C–25°C) for 5 min.
e. Place 1.5 mL LoBind Tube on magnet for 5 min.
f. Keeping tube on magnet, transfer the 400 μL supernatant (AbSeq PCR1 and Sample Tag products) to a new 1.5 mL tube without disturbing beads (mRNA targeted PCR1 products).
g. Store the supernatant at room temperature (15°C–25°C) while purifying and eluting the mRNA targeted PCR1 products (27a–27h below), then purify the AbSeq and Sample Tag PCR1 products.
27. Purifying mRNA targeted PCR1 products
a. Keeping tube on magnet, gently add 500 μL fresh 80% ethyl alcohol to the tube of SPRISelect beads bound with mRNA targeted PCR1 products and incubate 30 s. Remove supernatant.
b. Repeat step 27a once for two washes.
c. Keeping tube on magnet, use a small-volume pipette to remove residual supernatant from tube, and discard.
d. Air-dry beads at room temperature (15°C–25°C) for 5 min.
e. Remove tube from magnet and resuspend bead pellet in 30 μL of Elution Buffer (Cat. No. 91-1084). Vigorously pipette-mix until beads are uniformly dispersed. Small clumps do not affect performance.
f. Incubate at room temperature (15°C–25°C) for 2 min, and briefly centrifuge.
g. Place tube on magnet until solution is clear, usually ≤30 s.
h. Pipette the eluate (∼30 μL) into a new 1.5 mL LoBind Tube (purified mRNA targeted PCR1 products).

**Pause Point:** Store at 2°C–8°C before proceeding in ≤24 h or at –25°C to –15°C for ≤6 months.

28. Purifying combined AbSeq/Sample Tag PCR1 products
a. Pipette 100 μL SPRISelect beads into the tube with 400 μL AbSeq/Sample Tag PCR1 products from step 26g above. Pipette-mix 10 times.
b. Incubate at room temperature (15°C–25°C) for 5 min.
c. Place on magnet for 5 min.
d. Keeping tube on magnet, remove supernatant.
e. Keeping tube on magnet, gently add 500 μL fresh 80% ethyl alcohol, and incubate 30 s. Remove supernatant.
f. Repeat step 28e once for two washes.
g. Keeping tube on magnet, use a small-volume pipette to remove residual supernatant from tube, and discard.
h. Air-dry beads at room temperature (15°C–25°C) for 5 min.
i. Remove tube from magnet and resuspend bead pellet in 30 μL Elution Buffer (Cat. No. 91-1084). Vigorously pipette-mix until beads are uniformly dispersed. Small clumps do not affect performance.
j. Incubate at room temperature (15°C–25°C) for 2 min, and briefly centrifuge.
k. Place tube on magnet until solution is clear, usually ≤30 s.
l. Pipette the eluate (∼30 μL) into a new 1.5 mL LoBind Tube (purified AbSeq/Sample Tag PCR1 products).

**Pause Point:** Store at 2°C–8°C before proceeding in ≤24 h or at –25°C to –15°C for ≤6 months.

**Figure 8:**
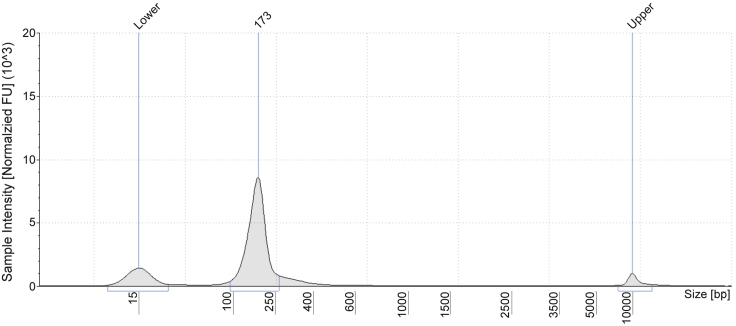
Example of AbSeq/Sample Tag PCR1 Products The largest peak should be approximately 170 bp. Other base pair lengths present are indicative of incomplete double-sided selection.

29. Quantify AbSeq/Sample Tag PCR1 products
a. Dilute an aliquot (∼2 μL) 1:1 with nuclease-free water, and run on the Agilent TapeStation with the High Sensitivity D5000 ScreenTape.
b. Measure the yield of the largest peak of the AbSeq/Sample Tag PCR1 products (∼170 bp, Figure 8).

**Table 6:** PCR1 Reaction Mix for Targeted mRNA, AbSeq, and Sample Tags

Component	1 Library (μL)	1 Library + 20% Overage (μL)	2 Libraries + 10% Overage (μL)
PCR MasterMix	100.0	120.0	220
Universal Oligo	20.0	24.0	44
Bead RT/PCR Enhancer	12.0	14.4	26.4
PCR1 primer panel	40.0	48.0	88
(Optional) PCR1 Panel Supplement	10	12	22
Sample Tag PCR1 Primer	1.2	1.4	2.64
BD AbSeq Primer	12.0	14.4	26.4
Nuclease-Free Water	Up to 14.8	Up to 17.8	Up to 32.6
Total	200.0	240.0	440

**CRITICAL:** Do not use fast cycling mode

**Table 7:** Thermocycler Program for PCR1

Step	Cycles	Temperature	Time
Hot Start	1	95°C	3 min
Denaturation	10–15	95°C	30 s
Annealing		60°C	3 min
Extension		72°C	1 min
Final extension	1	72°C	5 min
Hold	1	4°C	∞

**Table 8:** Suggested PCR1 Cycles for Given Cell Inputs

No. Cells Targeted on Cartridge	Suggested PCR Cycles for PCR1
500	15
1,000	14
2,500	13
5,000	12
10,000	11
20,000	10

c. Dilute an aliquot of AbSeq/Sample Tag PCR1 products to 0.1–1.1 ng/μL with Elution Buffer (Cat. No. 91-1084) before index PCR of AbSeq PCR1 products. Use undiluted AbSeq/Sample Tag PCR1 products for Sample Tag PCR2 amplification.
30. Perform PCR2 of targeted mRNA (Table 9) and Sample Tag products (Table 10). The AbSeq PCR1 products do not require additional amplification beyond index PCR.

a. In pre-amplification workspace, pipette reagents into a new 1.5 mL LoBind Tube on ice:

**Table 9:** Targeted mRNA PCR2 Reaction Mix

Component	1 Library (μL)	1 Library + 20% Overage (μL)	2 Libraries + 10% Overage (μL)
PCR MasterMix	25.0	30.0	55
Universal Oligo	2.0	2.4	4.4
PCR2 primer panel	10.0	12.0	22
(Optional) PCR2 Panel Supplement	2.5	3	5.5
Nuclease-Free Water	Up to 8.0	Up to 9.6	
Total	45.0	54.0	99

**Table 10:** Sample Tag PCR2 Reaction Mix

Component	1 Library (μL)	1 Library + 20% Overage (μL)	2 Libraries + 10% Overage (μL)
PCR MasterMix	25.0	30.0	55
Universal Oligo	2.0	2.4	4.4
Sample Tag PCR2 Primer	3.0	3.6	6.6
Nuclease-Free Water	18.0	9.6	39.6
Total	45.0	54.0	99

This reaction will further amplify the mRNA products. Add all components to a 1.5 mL LoBind tube on ice.

This reaction will amplify the Sample Tag products from PCR1. Combine all components from this table into a 1.5 mL LoBind tube on ice.

b. Gently vortex mix, briefly centrifuge, and place back on ice.
c. Bring PCR2 mixes into post-amplification workspace.
d. In two separate, new 0.2 mL PCR tubes:
i. mRNA targeted PCR1 products: Pipette 5.0 μL products into 45.0 μL mRNA targeted PCR2 reaction mix.
ii. Sample Tag PCR1 products: Pipette 5.0 μL products into 45.0 μL Sample Tag PCR2 reaction mix.
e. Gently vortex, and briefly centrifuge.
f. Program the thermal cycler (Table 11).

Program a thermocycler to this program without using fast cycling mode.

**Pause Point:** PCR2 products can be stored at 2°C–8°C for ≤24 h or stored at −25°C to −15°C for ≤6 months.

31. Purify targeted mRNA and Sample Tag PCR2 products
a. Vortex SPRISelect beads at high speed 1 min until beads are fully resuspended.
b. Briefly centrifuge PCR2 products.
c. To 50.0 μL PCR2 products pipette:
i. mRNA targeted PCR2 products: **40 μL** SPRISelect beads.
ii. Sample Tag PCR2 products: **60 μL** SPRISelect beads.
d. Pipette-mix 10 times, and incubate at room temperature (15°C–25°C) for 5 min.
e. Place tube on strip tube magnet for 3 min. Remove supernatant.
f. Keeping tube on magnet, gently add 200 μL fresh 80% ethyl alcohol into tube, and incubate 30 s. Remove supernatant.
g. Repeat step 31f once for two washes.
h. Keeping tube on magnet, use a small-volume pipette to remove residual supernatant from tube and discard.
i. Air-dry beads at room temperature (15°C–25°C) for 3 min.
j. Remove tubes from magnet, and resuspend bead pellet in 30 μL Elution Buffer (Cat. No. 91-1084). Pipette-mix until beads are fully resuspended.
k. Incubate at room temperature (15°C–25°C) for 2 min, and briefly centrifuge.
l. Place tubes on magnet until solution is clear, usually ≤30 s.
m. Pipette entire eluate (∼30 μL) of each sample into two separate new 1.5 mL LoBind Tubes (purified mRNA targeted PCR2 and Sample Tag PCR2 products).

**Pause Point:** Store at 2°C–8°C before proceeding on the same day or at –25°C to –15°C for ≤6 months.

n. Estimate the concentration of each sample by quantifying 2 μL of the PCR2 products with a Qubit™ Fluorometer using the Qubit dsDNA HS Assay Kit.
o. Dilute an aliquot of the products with Elution Buffer (Cat. No. 91-1084):
i. mRNA targeted PCR2 products: 0.2–2.7 ng/μL.
ii. Sample Tag PCR2 products: 0.1–1.1 ng/μL.
32. Perform index PCR to prepare final libraries
a. In pre-amplification workspace, prepare the three libraries + 20% overages of the final amplification mix for each of the three products. Pipette reagents into a new 1.5 mL LoBind Tube on ice (Table 12):

Combine all components in a 1.5 mL LoBind Tube on ice. Utilize a different reverse primer for each sample that will be run on the same sequencing flow cell. The mRNA, AbSeq, and Sample Tag libraries from the same cartridge should use the same reverse primer.

b. Gently vortex mix, briefly centrifuge, and place back on ice.
c. Bring index PCR mixes to post-amplification workspace.
d. In three separate, new 0.2 mL PCR tubes:
i. mRNA targeted PCR2 products: Pipette 3.0 μL of 0.2–2.7 ng/μL products into 47.0 μL index PCR mix.
ii. Sample Tag PCR2 products: Pipette 3.0 μL of 0.1–1.1 ng/μL products into 47.0 μL index PCR mix.
iii. AbSeq PCR1 products: Pipette 3.0 μL of 0.1–1.1 ng/μL products into 47.0 μL index PCR mix.
e. Gently vortex and briefly centrifuge.
f. Program the thermal cycler. (Tables 13 and 14)

Program a thermocycler to run this program. Refer to Table 14 for cycle numbers.

This table is a suggestion for the number of index cycles to use depending on the concentration of each library. It is possible you may need to adjust the number of cycles per experiment.

**Pause Point:** The Index PCR product can be stored at 2°C–8°C for ≤24 h, or at −25°C to −15°C for ≤6 months.

**Figure 9:**
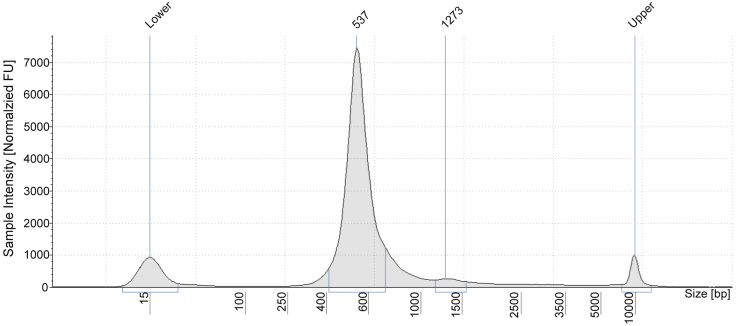
Example of Final mRNA Library TapeStation Trace The major peak length will depend on the PCR panels used. If you detect some transcripts with longer base pair lengths, this is typically not an issue as shorter transcripts amplify more efficiently than longer products.

**Figure 10:**
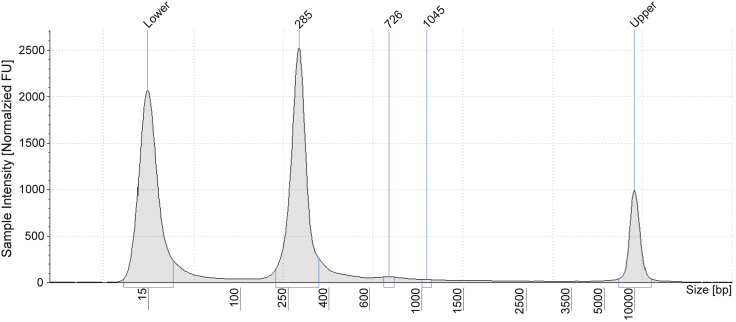
Example of Final Sample Tag Library TapeStation Trace The expected size of the final Sample Tag library is approximately 290 bp. If you observe a smaller peak (around 270 bp), it is likely those transcripts are AbSeq products.

**Figure 11:**
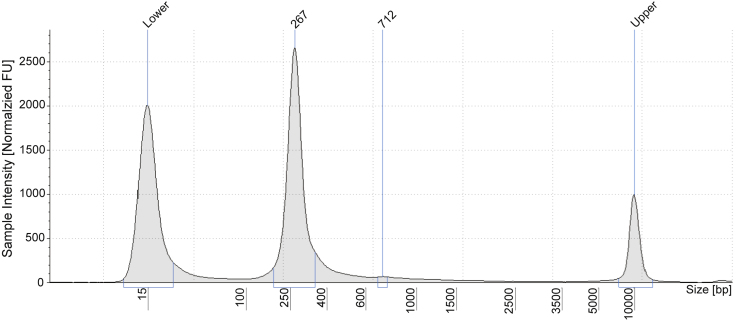
Example of Final AbSeq Library TapeStation Trace The expected size of the AbSeq final library is around 270bp.

33. Purify index PCR products
a. Vortex SPRISelect beads at high speed 1 min until beads are fully resuspended.
b. Briefly centrifuge index PCR products.
c. To 50.0 μL of each of the individual index PCR products pipette:
i. mRNA targeted library: **35 μL** SPRISelect beads.
ii. AbSeq and Sample Tag libraries: **40 μL** SPRISelect beads.
d. Pipette-mix 10 times. Incubate at room temperature (15°C–25°C) for 5 min.
e. Place each tube on strip tube magnet for 3 min. Remove supernatant.
f. Keeping tube on magnet, for each tube, gently add 200 μL fresh 80% ethyl alcohol into tube and incubate 30 s. Remove supernatant.
g. Repeat step 33f for a second wash
h. Keeping tubes on magnet, use a small-volume pipette to remove residual supernatant from tube, and discard.
i. Air-dry beads at room temperature (15°C–25°C) for 3 min.
j. Remove tubes from magnet and resuspend each bead pellet in 30 μL Elution Buffer (Cat. No. 91-1084). Pipette-mix until beads are fully resuspended.
k. Incubate at room temperature (15°C–25°C) for 2 min, and briefly centrifuge.
l. Place tubes on magnet until solution is clear, usually ≤30 s.
m. For each tube, pipette entire eluates (∼30 μL) into three separate new 1.5 mL LoBind Tubes (final sequencing libraries).
34. Perform quality control on the final sequencing libraries
a. Estimate the concentration of each sample by quantifying 2 μL of the final sequencing library with a Qubit Fluorometer using the Qubit dsDNA HS Kit, following manufacture’s protocol, to obtain an approximate concentration of PCR products to dilute for quantification on an Agilent 4200 TapeStation. Follow the manufacturer’s instructions. The expected concentration of the libraries is >1.5 ng/μL.
b. Measure the average fragment size of the mRNA targeted library by using the Agilent TapeStation with the High Sensitivity D5000 tape following the manufacturer’s instructions.
c. The final mRNA targeted library should show a fragment distribution that depends on the panel used (Figure 9). The expected size of Sample Tag index PCR product is 290 bp (Figure 10). You might observe a smaller peak of ∼270 bp, which corresponds to AbSeq product. The expected size of AbSeq index PCR products is ∼270 bp (Figure 11).

**CRITICAL:** Do not use fast cycling mode:

**Table 11:** Thermocycler Program for PCR2

Step	Cycles	Temperature	Time
Hot Start	1	95°C	3 min
Denaturation	10	95°C	30 s
Annealing		60°C	3 min
Extension		72°C	1 min
Final extension	1	72°C	5 min
Hold	1	4°C	∞

***Note:*** when preparing the libraries from two independent cartridges, prepare two separate master mixes with unique reverse primers. For information on ordering additional indices beyond the four indices included with the reagent kit contact scomix@bdscomix.bd.com.

**Table 12:** Indexing PCR Reaction Mix

Component	1 Library (μL)	For Three Libraries (mRNA, AbSeq, Sample Tag) + 10% Overage (μL)
PCR MasterMix	25.0	82.5
Library Forward Primer	2.0	6.6
Library Reverse Primer	2.0	6.6
Nuclease-Free Water	18.0	59.4
Total	47.0	155.1

**CRITICAL:** Do not use fast cycling mode:

**Table 13:** Thermocycler Program for Indexing PCR

Step	Cycles	Temperature	Time
Hot Start	1	95°C	5 min
Denaturation	6–8	95°C	30 s
Annealing		60°C	30 s
Extension		72°C	30 s
Final extension	1	72°C	1 min
Hold	1	4°C	∞

**Table 14:** Suggested PCR Cycle Numbers for Indexing PCR

Conc. Index PCR Input for Targeted mRNA Libraries (ng/μL)	Conc. Index PCR Input for Sample Tag and AbSeq Libraries 9 (ng/μL)	Suggested PCR Cycles
1.2–2.7	0.5–1.1	6
0.6–1.2	0.25–0.5	7
0.2–0.6	0.1–0.25	8

d. To calculate the concentration in nM, use the following equation:

C = X × 1×10^6^ × (1/MW) × (1/S)

Where

X = the concentration of the library calculated by Qubit in ng/μL

1 × 10^6^ is used to convert μL to L,

MW = the molecular weight of dsDNA

S = the average size of your library determined by the Tape Station or Bioanalyzer.

For example, if the Qubit reading for an mRNA library is 45 ng/μL with an average library size of 585 bp, the equation would be:

C = 45 ng/μL × 1×10^6^ μL/L × (1/660 mol/g) × (1/585 bp)

C = 116.6 nM

35. Combining libraries for sequencing (Multiplexing)
a. To determine how much of each sample to pool for sequencing, first dilute each sample to the recommended concentration for library pooling according to the Illumina guide (usually 1–4 nM, Table 15).

Suggested flowcell loading concentrations for libraries and PhiX for different Illumina systems.

**Table 15:** Sequencing Flowcell Loading and PhiX Concentration

Illumina System	Sequencing Flowcell Loading Concentration	Phix Concentration
MiSeq V2	6–10 pM	10%
MiSeq V3	6–10 pM	10%
MiniSeq High or Mid Output	1–1.5 pM	20%
NextSeq High or Mid Output	1–1.5 pM	20%
HiSeq 2500	7–15 pM	10%
HiSeq 3000/4000	3 nM	15%
NovaSeq 6000	2 nM	20%

b. Next determine how many reads each sample requires. To do this, multiply the target cell number by the desired number of reads per cell. For example, if you wanted to sequence sample one mRNA library at 5,000 reads per cell, and had a target number of 10,000 cells, the number of reads for the sample would be 5,000 × 10,000 = 5,000,000 reads.
c. Determine the fraction of the reads will go toward each sample. This can be done by dividing by each sample by the total reads needed across all samples. If you wanted to multiplex the Sample Tag, mRNA, and AbSeq libraries from a sample and each has 5,000,000 reads, 50,000,000 reads, and 150,000,000 reads respectively, the total reads is 205,000,000. The fraction of reads that will go to the Sample Tag is 0.02.
d. To determine the volume (μL) of each sample diluted to the flowcell loading concentration needed to pool for the final multiplexed library, multiply the fraction of reads by the total volume of the library needed. For example, to calculate the volume of Sample Tag (from step 35c) you need in your final library of 150 μL, multiply 0.02 by 150 to determine you will need 3.66 μL.

***Note:*** If the volume of library requires is prohibitively small a higher volume can be used for the pooling. For example, if only 5 μL is needed, a 100 μL pool can be made, and 5 μL of that pool can be taken into sequencing.

e. Pipette each required amount of sample into a LoBind 1.5 mL tube.
36. Sequence the libraries
a. Recommended sequencing depths are as follows:
i. mRNA: 5,000–10,000 reads per cell
ii. Sample Tag: 600–1,000 reads per cell
iii. AbSeq: 400–1,000 reads per cell per antibody

***Note:*** The recommended sequencing depths are provided as a starting point, since the number of reads that should be targeted is not a fixed value, but rather depends on several factors. First, the number of reads needed per cell for each mRNA library will depend on how many primers are included in your panel and the abundance of the transcripts of interest. If the goal is to capture rare transcripts as much as possible, a higher read per cell sequencing target may be beneficial. Similarly, the targeted reads per cell for each AbSeq library will depend on the makeup of the antibody panel. Proteins that are expressed at high levels will use proportionally more reads, which can leave other antibodies in the panel poorly resolved, particularly if they target proteins that are expressed at low levels.

### Primary Analysis Pipeline

**Timing: 1 h to upload data; run time varies**

37. Download fastq files from BaseSpace from the “File” menu using the BaseSpace Sequence Hub Downloader or obtain Fastq files from your sequencing core.
38. Create Seven Bridges login by going to http://www.sevenbridges.com/bdgenomics/
39. Create a new Project in Seven Bridges by clicking on the “Projects” tab and selecting “Create a project”.
40. Upload the fastq files using the Seven Bridges Uploader by clicking “Upload Files” and selecting the appropriate Fastq files (R1 and R2 are required.)

***Note:*** the uploader can be downloaded from the “Data Tools” section of the tab “Data”.

41. In the “Files” tab of the project, add the fasta reference files for the mRNA and AbSeq panels to the project
a. Generate AbSeq reference file by going to http://abseq-ref-gen.genomics.bd.com/ and selecting the antibodies that were used in your experiment. Use the resulting fasta-file and upload using the Seven Bridges Uploader.
b. Copy the appropriate mRNA fasta reference file (based on the gene panel that you used) to the project from the “Demo Project” in the “Projects” section of file addition
42. In the “Apps” section of the project, click on “add app” and select the “BD Rhapsody™ Targeted Analysis Pipeline” by clicking “run.
43. In the new screen, navigate to the “Inputs” section on the left hand side, select the AbSeq .fasta file as the “AbSeq Reference”, the .fastq files for the “Reads”, and the mRNA .fasta file as the “Reference”.
44. In the “App Settings” section select “Single-Cell Multiplexing Kit- Human” from the dropdown menu in “Multiplexing_Settings” to enable Sample Tag calling. Annotate the Sample Tags as appropriate for your experiment.
45. Select “Run” to trigger the analysis pipeline. Depending on the size of the experiment, the run can take several hours up to a day.
46. Once analysis is complete, output files will populate in the “Outputs” section of the analysis.
a. *“RSEC_MolsPerCell.csv”* and *“DBEC_MolsPerCell.csv”* files can be found in the “Data Tables” section.

***Note:*** RSEC_MolsPerCell file can be used regardless of sequencing depth. DBEC_MolsPerCell file should be used if the mean RSEC sequencing depth is at least 6. When comparing files from two different pipeline outputs, RSEC file should be used unless all genes being compared underwent the same correction (either RSEC or DBEC). This information can be found in the #Targets# section of the Metrics Summary*.*

b. *“Sample_Tag_Calls.csv”* can be found in the “Multiplex” section.
c. Zip files containing the RSEC_MolsPerCell files for only the cells associated with each Sample Tag can be downloaded from the “Multiplex” section.

## Expected Outcomes

Type of data that are generated: The protocol presented here details sample and library preparation for the detection of targeted transcript and surface protein expression at the single-cell level. After sequencing and pre-processing of the Fastq files, the final output will essentially yield a large and sparse data matrix containing the molecule counts for mRNA and the proteins for each cell (MolsPerCell.csv).

For the analysis of these complex data sets, there are different options available. Users that prefer a graphical user interface can use commercial software packages such as SeqGeq or BioTuring (among others). If more versatile options are required it is recommended to use the R environment and dedicated packages for single-cell analysis which are commonly available on Bioconductor. A full description of this process and all available analysis approaches is beyond the scope of this protocol and we refer the reader to recent reviews which also include excellent online tutorials and links to additional resources (Amezquita et al., 2020; Luecken and Theis, 2019).

The exact number of cells that are called in the sequencing data will be less than the number of cells loaded on the cartridge. The number depends on the sample type, accuracy of cell counts and viability of the cells loaded. For a typical experiment with human leukocytes, after quality control filtering and removal of multiplets, users can expect to recover data for about 60% of the total cells loaded.

The final number of gene targets varies depending on the design of the target transcriptomic assay but can be up to 499 target genes. We reported that with an assay targeting 492 genes, 2,000–4,000 reads per cell from the transcriptomic portion of the library delivered sufficient resolution^1^. Like the transcriptomic analysis, the number of proteins targeted can be customized for specific experimental aims. We previously reported that with an assay targeting 41 proteins, read depths of 200–400 reads/antibody/cell from the protein portion of the library results in sufficient resolution^1^.

## Limitations

### Limitations to Assessing Protein Expression

Antibody-based probes are powerful tools for detection of specific protein targets. However, meaningful interpretation of sequencing-based protein measurements requires rigorous validation. In some instances we observed that the same antibody clone can yield different expression patterns in sequencing-based analysis relative to conventional cytometry (Mair et al., 2020). Furthermore, the sequencing read depth in context of the chosen antibody panel is of particular importance here. Highly expressed proteins (e.g., lineage markers such as CD4, CD8, HLA-DR) will use a significant portion of the sequencing reads, which may make it difficult to resolve less abundant proteins (such as IL-7Rα) if the chosen read depth is too low. While there is no general consensus yet for a minimum read depth in all experimental settings, it is recommended to calculate with at least 200–400 reads per antibody per cell as a rule of thumb (Mair et al., 2020).

### Limitations to Assessing Transcript Expression

Our previous comparison between whole transcriptome and targeted transcript analysis demonstrated that targeted analysis can be highly sensitive in detection of low-abundance transcripts, but some genes may be under-represented compared to whole transcriptome analysis (and vice versa). This may result from different amplification efficiencies between multiplexed targeted primers used in the Rhapsody platform and the template-switch process in other WTA platforms. A lack of a signal for a specific transcript (by either WTA or targeted transcriptomics) cannot necessarily be interpreted as absence of transcript expression. Further, transcripts that contain internal poly A stretches can also artificially inflate the number of captured transcript molecules with WTA that would otherwise correctly not be captured in a 3’ end targeted approach. Differences in poly A site expression (location and total number) due to tissue types and cell states (i.e., activated versus resting) can cause inherent heterogeneity in abundance and identity of observed transcripts. Because of these issues one possible strategy is to first use WTA on the sample of interest to inform locations of poly A sites to include in the primer design of targeted mRNA panels to better correlate these two types of data. Finally, it is important to remember in this context that the dynamic range of expression for proteins spans about 6–7 orders of magnitude, whereas transcript copy numbers span about two orders of magnitude (Azimifar et al., 2014; Schwanhausser et al., 2011), with a mean copy number of ∼4 copies per cell, which are inherently difficult to detect with any single-cell analysis approach.

### Limitations to the Number of Cells that Can Be Analyzed and Enrichment Strategies

If the experimental goal is an exploratory snapshot of all immune cells, one may start with a bulk immune cell population (e.g., human peripheral blood lymphocytes). However, if a specific and possibly rare cell subset is of interest, then enriching for these cells may be necessary to obtain meaningful data. For example, human dendritic cell subsets might represent only 10–100 cells in a typical PBMC sample of 20,000 cells, thus requiring enrichment for meaningful analysis.

If enrichment by cell sorting (FACS) is performed, it is important to ensure that the used fluorochrome-conjugated antibodies do not overlap or interfere with the antibody clones used in the downstream oligo-antibody assay. This can be tested e.g., by prior co-staining of different antibody clones by flow cytometry and selecting only compatible clones. As an alternative, if the same clones have to be used, it is possible to evaluate the cell staining ratios of fluorochrome-conjugated antibodies with AbSeq antibodies coupled with complementary sequence-conjugated fluorochrome (Flow Proxy) to determine an optimal ratio for cell sorting while maintaining AbSeq staining. In these scenarios, fluorochrome-conjugated Abs can be stained simultaneously with oligo-conjugated AbSeq reagents saving researchers time and minimize handling and potential perturbations to cells.

Importantly, if magnetic enrichment protocols are used, positive selection magnetic bead enrichment should be avoided while negative selection/depletion is preferred. Ferrous particles that remain on cells from enrichment may interfere with downstream processing in the nano-well-cartridge.

## Troubleshooting

### Problem 1

Final library concentration lower than what is required for sequencing.

### Potential Solution

If the final library has too low of a concentration, the indexing PCR can be re-done with the PCR2 products using more than the recommended 6–8 cycles. The additional number of cycles will depend on how much more the final library concentration needs to increase. It is recommended that you start with just one or two extra cycles more to avoid as much PCR amplification bias as possible. Another option is to return to PCR1 products, or completely remake the library from the saved exonuclease treated beads.

### Problem 2

**Figure 12:**
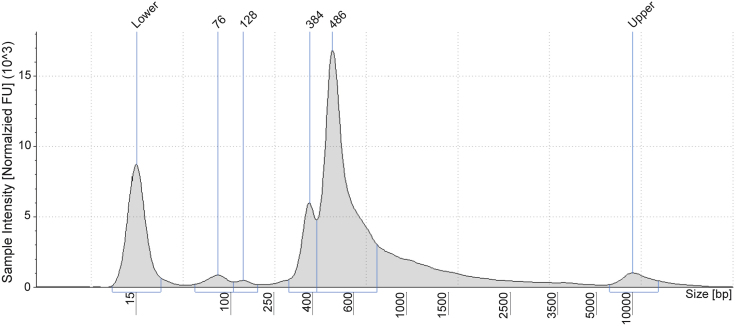
Example of a Poor-Quality Trace This is an example of a library that had an incomplete double-sided selection. The library should undergo another round of cleanup.

Tape Station traces are showing peaks that are not very clean (see Figure 12).

### Potential Solution

This is generally due to poor SPRISelect bead clean up. Ensure you are using exactly the indicated volume of beads and there are no SPRISelect bead droplets on your pipette tips. The SPRISelect bead clean up can be repeated on a sample that has already been cleand up, using the same ratio of beads to sample as before, although some sample loss should be expected.

### Problem 3

Visualization of AbSeq data in FlowJo does not show the expected expression patterns.

### Potential Solution

One of the possible analysis options is to convert the molecule per cell CSV-file into the flow cytometry standard (FCS) file format by using R packages such as flowCore or premessa. This allows the visualization of bivariate plots in the popular software package FlowJo. However, the data ranges for RNA and protein expression are widely different, and thus it is important to adjust the transformation of these two molecule classes appropriately. One option is to choose the arcsinh transform in FlowJo and adjusting the co-factor as required to achieve bimodal expression for well-defined protein markers (e.g., CD3).

### Problem 4

No signal obtained for oligo-antibody conjugate after prior enrichment with cell sorting.

### Potential Solution

The reason for this can be that a competing fluorescent-labeled antibody clone was used for cell enrichment, preventing the subsequent binding of the oligo-antibody conjugate to its target. As discussed in more detail in the section “Limitations”, if the same antibody targets are needed for enrichment as well as for readout during the multi-omic workflow, it is recommended to use different antibody clones. Experimenters need to perform prior co-staining experiments using these clones in a conventional flow cytometry experiment to test whether the clones are compatible (e.g., for targeting human CD3, the two clones OKT3 and SK7 can be used).

### Problem 5

Poor resolution obtained for oligo-antibody signal.

### Potential Solution

For some antibodies and experimental setups, the final concentration as pre-determined by the manufacturer might be suboptimal. In this case, it is recommended to titer the oligo-antibody conjugate on a conventional flow cytometer using an oligo-nucleotide coupled to fluorochromes (oligo-dT-fluorochrome).

## Resource Availability

### Lead Contact

Further information and requests for resources and reagents that are not commercially available should be directed to and will be fulfilled by the Lead Contact, Martin Prlic (mprlic@fredhutch.org).

### Materials Availability

This study did not generate new unique reagents.

### Data and Code Availability

The published article includes all code generated during this study. Optional code for data processing post Seven Bridges (not covered in this protocol) are available at GitHub: https://github.com/MairFlo/Targeted_transcriptomics
